# Drug sensitivity and genome-wide analysis of two strains of *Mycoplasma gallisepticum* with different biofilm intensity

**DOI:** 10.3389/fmicb.2023.1196747

**Published:** 2023-08-09

**Authors:** Xiaoyan Ma, Li Wang, Fei Yang, Jidong Li, Lei Guo, Yanan Guo, Shenghu He

**Affiliations:** ^1^Clinical Veterinary Laboratory, Institute of Animal Science and Technology, Ningxia University, Yinchuan, China; ^2^Ningxia Xiaoming Agriculture and Animal Husbandry Co., Ltd., Yinchuan, China; ^3^Ningxia Academy of Agriculture and Forestry Sciences, Yinchuan, China

**Keywords:** *Mycoplasma gallisepticum*, biofilm, formation, drug sensitivity, whole-genome analysis

## Abstract

*Mycoplasma gallisepticum* (MG) is one of the major causative agents of chronic respiratory diseases in poultry. The biofilms of MG are highly correlated to its chronic infection. However data on genes involved in biofilm formation ability are still scarse. MG strains with distinct biofilm intensity were screened by crystal violet staining morphotyped and characterized for the drug sensitivity. Two MG strains NX-01 and NX-02 showed contrasted ability to biofilm formation. The biofilm formation ability of NX-01 strain was significantly higher than that of NX-02 strain (*p* < 0.01). The drug sensitivity test showed that the stronger the ability of MG stain to form biofilms, the weaker its sensitivity to 17 antibiotic drugs. Moreover, putative key genes related to biofilm formation were screened by genome-wide analysis. A total of 13 genes and proteins related to biofilm formation, including *ManB*, *oppA*, *oppD*, *PDH*, *eno*, *RelA*, *msbA*, *deoA*, *gapA*, *rpoS,* Adhesin P1 precursor, S-adenosine methionine synthetase, and methionyl tRNA synthetase were identified. There were five major discrepancies between the two isolated MG strains and the five NCBI-published MG strains. These findings provide potential targets for inhibiting the formation of biofilm of MG, and lay a foundation for treating chronic infection.

## 1. Introduction

*Mycoplasma gallisepticum* (MG) is one of the important pathogens affecting poultry (Ghanem et al., 2017). It is widely distributed in all poultry countries worldwide, causing serious economic losses to the global poultry industry (Roussan et al., 2015; Feberwee et al., 2021; Marouf et al., 2022). MG mainly causes chronic respiratory diseases in chickens and other poultry and infectious sinusitis in turkeys (Sulyok et al., 2019). In addition, the infection of MG can cause low carcass grading and feed conversion efficiency of broiler chickens. With the increase of the weak brood rate, the egg production and egg hatching rate of laying hens tend to decrease (Beaudet et al., 2019).

Many therapeutic drugs have been developed for MG infection, such as macrolides, quinolones, tylomycin, and gentamicin (McAuliffe et al., 2006; Abd El-Hamid et al., 2019). However, the drug resistance of MG is becoming increasingly severe (Beylefeld et al., 2018), and biofilms may be one of the main causes. Bacterial biofilm plays an important role in the bacterial disease process, allowing bacteria to evade the host’s immune defenses, inducing drug resistance and increasing toxin accumulation (Sorci, 2013). The formation of biofilm poses significant clinical challenges and exacerbates the issues of drug resistance and bacterial virulence (Tassew et al., 2017; Feng et al., 2021). Studies have shown that the development of a biofilm by MG could contribute to persistent infections, rendering the eradication of MG challenging (Feng et al., 2020). The complete characterization of MG biofilm formation is key in assessing and combating resistance to infectious poultry diseases, particularly in commercial farming (Taylor-Robinson and Bébéar, 1997). Despite the wealth of information and corresponding treatment modalities available for biofilm formation in bacteria and other mycoplasma (Stewart, 2002; Sharma et al., 2019), there is a lack of research on MG biofilm.

*Mycoplasma gallisepticum* is a prokaryote intermediate between bacteria and viruses and lacks a cell wall (Ishfaq et al., 2020). A few key genes regulate the formation and attachment of bacterial biofilms to surfaces (Parker et al., 2009; Liu et al., 2022). For example, the formation of *Streptococcus gordonii* biofilm depends on arginine, which regulates the development of *Streptococcus gordonii* biofilm mainly by activating the regulatory pathway of the ArcR gene (Robinson et al., 2018). In addition, the *ssrS* gene of *Haemophilus parasuis* is associated with the antitoxin system, regulating biofilm formation and cell persistence (Jiang et al., 2021). Several genes that have a crucial role in the biofilm formation of various strains have been well-documented. However, there is a significant paucity of research on MG. So far, only a few articles have shown that *ManB*, *VlhA*, *PDH*, ABC transporter penetrase, ABC transporter ATP-binding protein, and other genes may be involved in forming MG biofilm (Chen et al., 2012; Wang et al., 2017). It is very important to study the regulatory genes of biofilm formation for controlling MG chronic infection. Genomic analyses can help elucidate genes functionalities in MG as recently documented (Semashko et al., 2022). Such approach could help to elucidate putative genes involved in biofilm formation ability and drug resistance.

The objective of this study was to screen MG strains with contrasted ability in terms of biofilm formation in Ningxia Hui Autonomous Region in China and study their drug sensitivity. It also aimed to gaining insights in the putative genes involved in biofilm formation ability using a genome-wide analysis on both strains having contrasted ability to biofilm formation.

## 2. Materials and methods

### 2.1. Main materials

The improved Frey culture medium was obtained from China Sea Biotechnology Co., LTD., Beijing, China. The bacterial genome DNA extraction kit was purchased from Tiangen Biochemical Technology Co., LTD., Beijing, China. Crystal violet was acquired from Chongming County Yuxi Reagent Factory. Ethanol (95%) and methanol were purchased from Guangnuo Chemical Technology Co., LTD., Shanghai, China. Glutaraldehyde fixative (2.5%, for electron microscopy) was from Proanti Biotechnology Development Co., LTD., Shaanxi, China. Ofloxacin, Ennofloxacin, Norfloxacin, Taymycin, Erythromycin, Talomycin, Temicoxacin, Spiramycin, Tetracycline Hydrochloride, Lincomycin, Gentamicin, Dakanamycin, Kanamycin, Streptomycin, Doxycycline, and Warnemylin were purchased from Golden Clonix Biotechnology Co., LTD., Beijing, China.

### 2.2. Bacterial strain isolation

Ethics approval and specific permission were not required for the study, as all samples were collected by the authors during necropsies with the consent of the owners. Air sacs of chickens suspected to be infected with *M*. *gallisepticum* were collected from poultry farms in Ningxia (Felice et al., 2020; Yadav et al., 2022). They were cut and placed in modified Frey’s liquid medium overnight at 4°C under sterile conditions. After filtration, they were inoculated into Frey liquid medium and incubated in a biochemical incubator at 37°C. The MG were purified by Kahya et al. (2010) and Sprygin et al. (2010), the harvested isolates were coated in the modified Frey’s solid medium and placed in a 5% CO_2_ incubator at 37°C for 3–7 days. Single colonies were selected and cultured in the modified Frey’s liquid medium, and the purified isolates were repeated three times to get the purified isolates. The colony morphology on the solid medium was observed with an inverted microscope.

Bacterial genomic DNA was extracted using the kit of Tiangen Biochemical Technology Company (Ahmad et al., 2020; Li L. et al., 2020). After the expanded culture, put 1 mL of fresh bacterial solution into a centrifuge tube and add 20 μL of protease K solution. Add 220 μL anhydrous ethanol and mix thoroughly. Then, the solution and precipitate are added to the adsorption Add 220 μL anhydrous ethanol and mix thoroughly. Then the solution and precipitate are added to the adsorption column, and 500 μL buffer GD is added to the column. Discard the waste liquid after centrifugation. Add 600 μL PW to the adsorption column, and the waste liquid was discarded after centrifugation. Add 50 μL eluent buffer TE in the middle of the adsorbed strain and let it stand at room temperature for 5 min. The solution was collected into the centrifuge tube to obtain the genome extract of MG strains. Meanwhile the isolated MG strain was amplified by PCR based on the OIE 16S rRNA and mgc2 primers to identify the MG strain in the bacterial solution (Table 1), nuclease-free water was used as a negative control in all PCR assays. Finally, the bacterial solution was mixed at a ratio of 1:1 to glycerol and stored at −80°C.

**Table 1:** List of *M*.*gallisepticum* strains PCR primer information.

Gene	primer	Sequence (5′-3′)	Stripe/bp	Tm/°C
16S rRNA	MG-F	GAGCTAATCTGTAAAGTTGGTC	183	55°C
	MG-R	GCTTCCTTGCGGTTAGCAAC		
mgc2	MG-F	CGCAATTTGGTCCTNATCCCCAACA	236–302	54°C
	MG-R	TAAACCCRCCTCCAGCTTTATTTCC		

### 2.3. Determination of biofilm formation capacity

Modified tests using crystal violet staining were used to quantify biofilm formation (Hu et al., 2010). The suspension concentration of the prepared MG strains was adjusted to 1 × 10^7^ CCU/mL, and 200 μL was inoculated on sterile 96-cell plates. Six replicates of the bacterial culture were prepared and subsequently incubated at 37°C for 12, 24, 36, and 48 h. Meanwhile, the crystal violet staining method was employed to measure the OD_590_ value of MG biofilm by enzyme marker, and the absorbance results were recorded. According to ODc value (ODc is equal to the average OD value of blank well), biofilm classification: OD ≤ ODc, no membrane capacity (−); ODc < OD ≤ 2ODc, weak biofilm forming ability (+); 2ODc < OD ≤ 4ODc, medium biofilm forming ability (++); and 4ODc < OD, strong biofilm forming ability (+++).

### 2.4. Determination of matrix growth curves of biofilm models *in vitro*

The growth curves of the biofilm matrix were measured by a semi-quantitative adhesion test (Liang et al., 2019). 200 μL of 1 × 10^6^ CCU/mL bacterial suspension was inoculated on 96-cell plates with six replicates per strain, after which the plates were then incubated at 37°C for 0, 3, 6, 9, 12, 24, 36, 48, and 72 h. In the corresponding period time, crystal violet staining was used to detect the OD_590_ value of two MG biofilms. According to the absorbance results, the biofilm matrix growth curve was drawn.

### 2.5. Observation of biofilm morphology by scanning electron microscopy

*Mycoplasma gallisepticum* strains were inoculated into the liquid Frey medium for 12 h, and the suspension concentration was adjusted to 1 × 10^4^ CCU/mL. The sterile six-well plate was used to hold the 14 mm round slip-plate, with 5 mL of bacterial suspension added to each well. The culture was incubated at 37°C for 24 h, and 1 mL 2.5% glutaraldehyde fixed solution (EM Grade) was added to fixed at 4°C overnight. The gradient elution was performed on each well was gradient eluted with ethanol of different concentration (30, 50, 70, 80, 90, and 95%). The obtained sample was sliced and photographed using an ISI-SX-40 scanning electron microscope (Shatila et al., 2021).

### 2.6. Drug sensitivity test

The drug sensitivity of the isolated stains were determined by Hannan’s microdilution method (Hannan, 2000). The suspension concentration of MG strains was adjusted to 1 × 10^4^ CCU/mL. 40 μL 512 μg/mL of antibiotics were added to the first hole of the 96-well plate, and add 20 μL sterile PBS from well 2 to 12. Then 20 μL of liquid was discarded from well 1 to 2 and mixed, and the same operation was performed until well 12. Thus, the antibiotics were continuously diluted at multiple ratios. Finally, 180 μL diluted MG bacterial solution was added to well 1–12. Growth control wells (containing culture medium and bacterial solution) and negative control Wells (containing liquid culture medium) were also set up and each antibiotic were repeated three times, After being inoculated, the 96-well plates were transferred to a constant temperature incubator set at 37°C for a week. The color of the growth control well was yellow, and the negative control well was pink. The highest concentration of the drug with no color change in the test well was considered the lowest inhibitory concentration of the drug (Gautier-Bouchardon, 2018).

### 2.7. Genome-wide sequencing and quality control

Sangon Bioengineering (Shanghai) Co., LTD. completed the genome-wide sequencing of the MG strains. MG complete genome sequences were obtained using Illumina HiSeq platform (Caporaso et al., 2012) for MG genome sequencing. The original image data file of the MG genome was processed by Base Calling and stored in FASTQ (Brown et al., 2017) file format. Next, the FastQC software was used to evaluate sequencing data quality. Using Trimmomatic (Bolger et al., 2014) was used to clear joint sequences and lower quality sequences to ensure that all the clean reads false discovery rate was <0.01.

### 2.8. Genome splicing and component analysis and gene function annotation

To splice sequencing data, the SPAdes (Bankevich et al., 2012) was utilized (Table 2). In order to fill gaps in the resulting contigs, the GapFiller (Boetzer and Pirovano, 2012) was employed. The accuracy of the spliced sequences was improved using PrInSeS-G (Massouras et al., 2010) correction tool that can rectify splicing errors during editing and detect small insertion losses. The genetic elements were predicted using Prokka (Seemann, 2014), and repeated sequences in the genome were identified using RepeatMasker (Saha et al., 2008). The CRISPRs were predicted through CRT (Bland et al., 2007) analysis. To obtain functional annotation information, gene sequences were analyzed separately using NCBI NR (Yu and Zhang, 2013), COG (Tatusov et al., 2000), GO (The Gene Ontology, 2019), and KEGG (Kanehisa and Goto, 2000), VFDB (Chen et al., 2016), CARD (McArthur et al., 2013), and CAZy (Lombard et al., 2014), and compared using NCBI Blast + (Altschul et al., 1997).

**Table 2:** List of bioinformatics tools and databases for whole genome sequencing of *M*.*gallisepticum*.

Name	URL	Edition
Illumina HiSeq platform	https://www.illumina.com.cn/	/
FASTQ	https://www.bioinformatics.babraham.ac.uk/projects/fastqc/	v0.11.2
Trimmomatic	http://www.usadellab.org/cms/index.php?page=trimmomatic	v0.36
SPAdes	http://bioinf.spbau.ru/spades	v3.5.0
GapFiller	http://www.baseclear.com/bioinformatics-tools/	v1.11
PrInSeS-G	https://updeplasrv1.epfl.ch/prinses/	v1.0.0
Prokka	http://vicbioinformatics.com/	v1.10
RepeatMasker	www.repeatmasker.org	v4.0.5
NCBI Blast +	https://blast.ncbi.nlm.nih.gov/Blast.cgi	v2.2.28
MEGA6	https://mega6.com.uy/Mega6/inicio	v6.0
CRT	http://www.room220.com/crt	v1.2
MUMmer	https://mummer4.github.io/	v4.0.0
NCBI NR	http://ncbi.nlm.nih.gov/	/
COG	https://www.ncbi.nlm.nih.gov/COG/	/
GO	http://www.geeontology.org	/
KEGG	http://www.kegg.jp	/
VFDB	http://www.mgc.ac.cn/VFs/	/
CARD	https://card.mcmaster.ca/	/
CAZy	http://www.cazy.org/	/
NCBI	http://ncbi.nlm.nih.gov/	/

### 2.9. Genome-wide comparative analysis

The annotation data of the genome-wide of the locally isolated MG strains were analyzed, including total gene length, GC content, coding gene, etc. Molecular evolutionary tree were constructed using N-J method and MEGA6 software (Tamura et al., 2013). The whole-genome sequences of MG strain were compared with those of the five MG strains available in the NCBI database. Subsequently, the linear analysis of genome-wide sequences of MG strains were conducted using the MUMmer (Delcher et al., 2003; Table 3). Finally, a plot was created to visualize the region of genetic differences.

**Table 3:** Strains of *Mycoplasma gallisepticum* for genome alignment.

Organism	Strain	RefSeq	Source area
*Mycoplasma gallisepticum*	NX-01	PRJNA972534	China
	NX-02	PRJNA972540	China
	NCTC10115	NZ_LS991952.1	Britain
	KUVMG001	NZ_CP070622.1	South Korea
	6/85	NZ_CP044224.1	United States
	mx-4	NZ_CP044226.1	United States
	f99 lab strain	NZ_CP028146.1	United States
	ts-11	NZ_CP044225.1	United States
	k5234	NZ_CP092251.1	United States
	CA06_2006.052-5-2P	NC_018412.1	United States
	S6	NC_023030.2	Russia
	R(low)	NC_004829.2	United States
	F	NC_017503.1	United States

## 3. Results

### 3.1. The bacterial strains showed contrasted ability in biofilm formation

**Figure 1:**
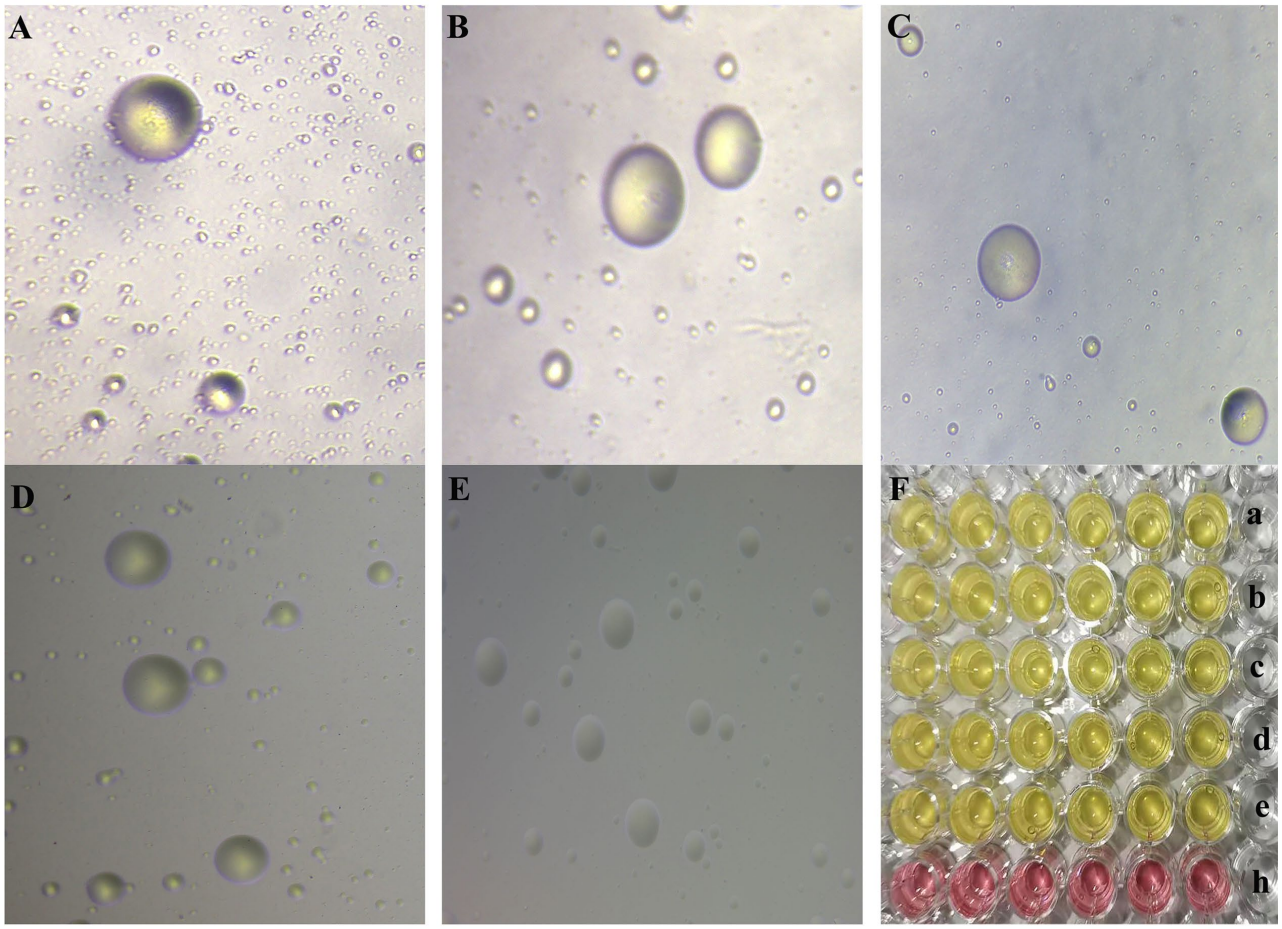
**(A–E)** The morphology of MG NX-01, NX-02, NX-03, NX-04, and NX-05 strains in modified solid medium under inverted microscope (10×), respectively. Consistent with the typical “fried egg” -like appearance of mycoplasma. **(F) a**–**e** was the color change of MG NX-01, NX-02, NX-03, NX-04, and NX-05 strains cultured in modified Frey liquid medium for 2–3 days, and **h** was the blank control group. Consistent with the characteristics of mycoplasma.

**Figure 2:**
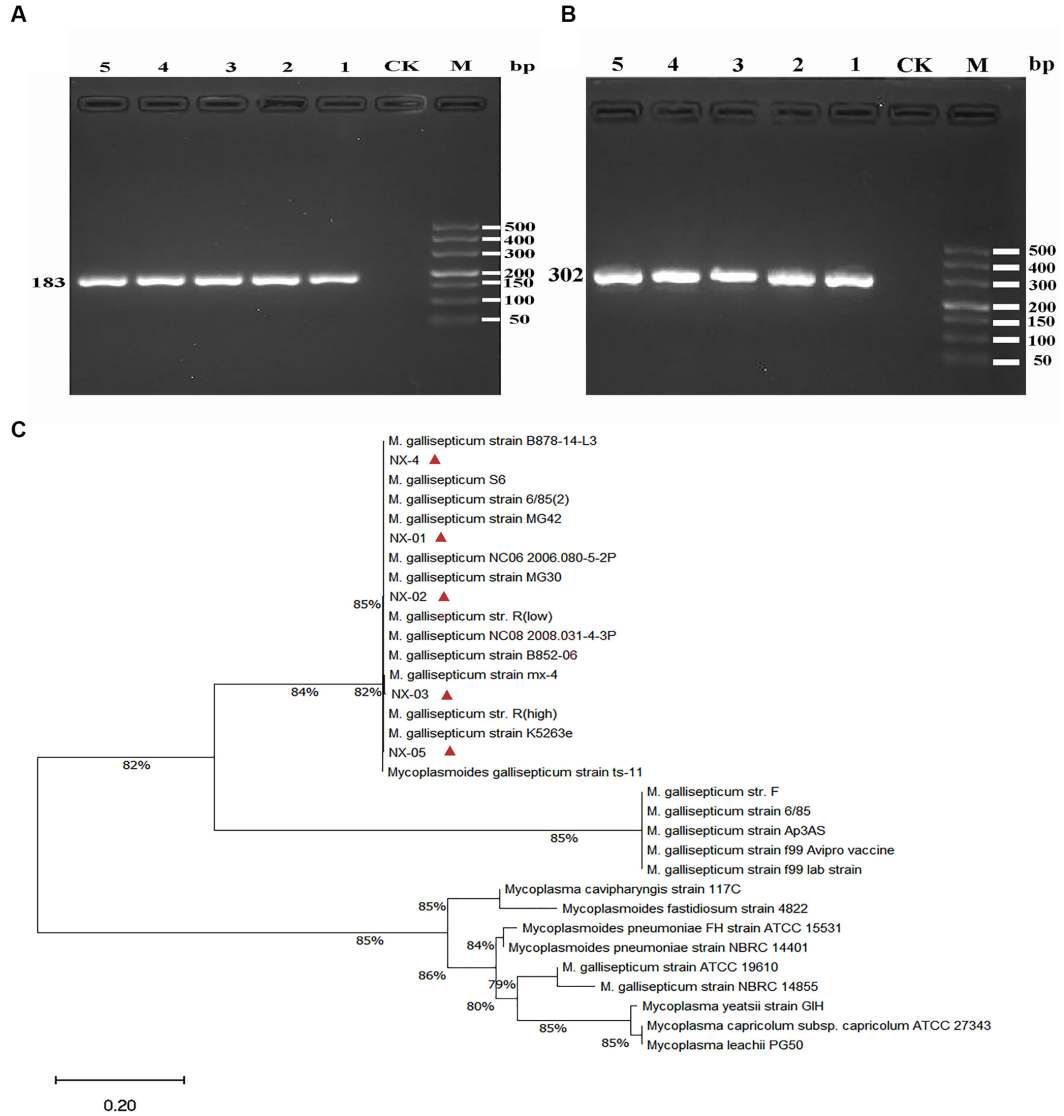
**(A)** The PCR results of MG 16S rRNA isolated from five strains. The fragment size was consistent with the expected size, indicating that the fragment was amplified correctly. **(B)** The PCR results of MG mgc2 isolated from five strains. The fragment size was consistent with the expected size, indicating that the fragment was amplified correctly. **(C)** The 16S rRNA phylogenetic tree of five MG isolates. The results showed that five MG strains were closely related to *Mycoplasma gallisepticum* uploaded by other NCBI. The above results indicated that the isolated strain was *Mycoplasma gallisepticum*.

**Figure 3:**
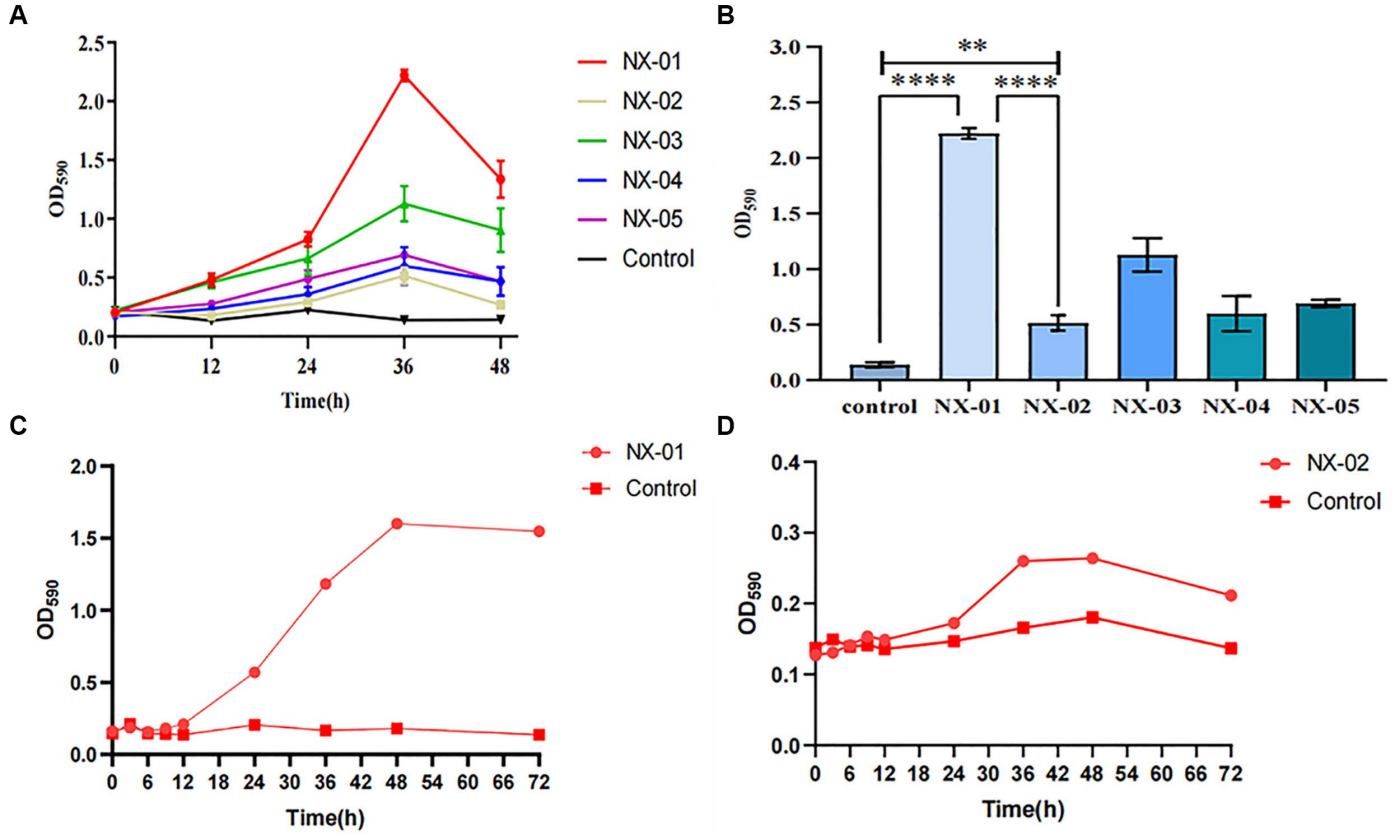
**(A)** Biofilm formation in different strains of MG. The data are means from three independent experiments. The *Mycoplasma gallisepticum* strains NX-01 and NX-03 were determined to be strong biofilm producers. The *M*. *gallisepticum* strain NX-05 was determined to be a medium biofilm producer. The *M*. *gallisepticum* strains NX-02 and NX-04 were determined to be weak biofilm producers. **(B)** At 36 h of biofilm culture, the OD_590_ readings of the *M*. *gallisepticum* strain NX-01 were significantly different from the other four strains. **(C)** Biofilm growth curve of *M*. *gallisepticum* strain NX-01. **(D)** Biofilm growth curve of *M*. *gallisepticum* strain NX-02.

According to strain isolation and identification, five MG strains NX-01, NX-02, NX-03, NX-04, and NX-05 were isolated during this study (Figures 1, 2). The capacity of biofilm formation showed that all 5 MG strains could form biofilms, with the following formation ability: NX-01 > NX-03 > NX-05 > NX-04 > NX-02 (Figure 3A), whereas the *M*. *gallisepticum* strains NX-01 and NX-03 showed strong biofilm forming ability (+++). Meanwhile, *M*. *gallisepticum* strain NX-05 showed medium biofilm-forming ability (++), and NX-04 and NX-02 were weak biofilm formers (+). The OD_590_ readings of the strong biofilm-forming strain NX-01 significantly differed from the medium and weak biofilm-forming strains at 12, 24, and 48 h of biofilm culture (*p* < 0.01). Additionally, at 36 h of biofilm culture, the OD_590_ readings of the *M*. *gallisepticum* strain NX-01 were significantly different from the other four strains (*p* < 0.01; Figure 3B). The results showed that NX-01 strain was the strongest strain and NX-02 strain was the weakest among the five MG strains. Therefore, these two strains were selected as the subsequent test bacteria.

The matrix growth curve test of the biofilm model *in vitro* showed that the biofilm formation of the tested MG was divided into the initial adhesion stage, aggregation stage, and biofilm maturation stage. The two tested strains NX-01 and NX-02reached the initial adhesion stage after 12 h culture.

The growth rate of the NX-01 strain was the highest at 12–48 h, and the strain gathered to form microcolonies, corresponding to the aggregation stage of the biofilm. There was no significant change in the number of viable cells of stain at 48–72 h (*p* > 0.05), corresponding to the maturation stage of the biofilm, at which the microcolony continued to expand and formed a mature biofilm with a dense structure (Figure 3C).

The cells of the NX-02 strain adhered to each other and gathered to form microcolonies within 12–36 h, after which they rapidly multiplied and began to adhere to microplates. During the logarithmic phase of biofilm growth from 36 to 42 h, the formed strain microcolonies fused with each other and developed into biofilm with a mature three-dimensional structure. At 48–72 h, bacterial metabolic activity in the biofilm decreased (Figure 3D). The results showed that the membrane growth rate of strong membrane-forming MG strain NX-01 was faster than that of weak membrane-forming strain (*p* < 0.01), and the maintenance time of biofilm of NX-01 strain was longer than that of NX-02 strain (*p* < 0.05).

**Figure 4:**
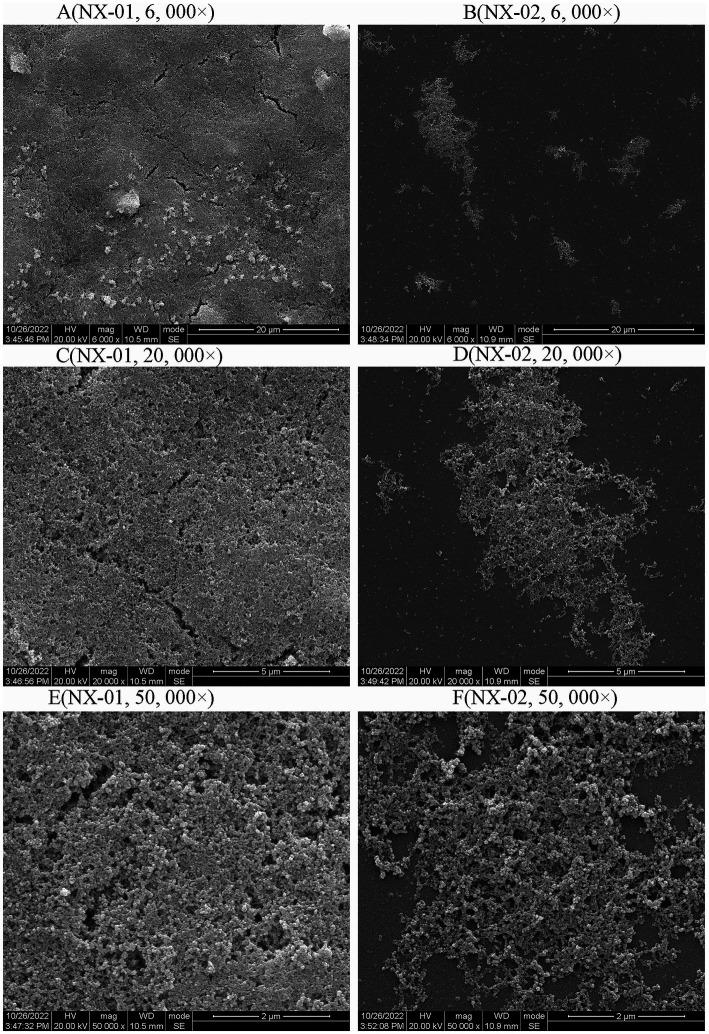
Scanning electron microscopy (SEM) analysis of the biofilm 0f NX-01 and NX-02 at gathering period. **(A)** MG NX-01(6,000×), **(B)** NX-02 (6,000×), **(C)** MG NX-01 (20,000×), **(D)** NX-02 (20,000×), **(E)** MG NX-01 (50,000×), and **(F)** NX-02 (50,000×).

Scanning electron microscopy (SEM) was used to visually detect the morphology of the biofilm adhesion stage of NX-01 and NX-02 strains. Bacterial aggregates of NX-01 strain was detected across the entire visual field at varying magnifications (Figure 4A). They formed the mature biofilm, which is closely adhered together after being wrapped by the extracellular polysaccharide and other substances secreted by pathogenic bacteria. The three-dimensional mushroom-like structure can even be observed at 50,000× magnification (Figure 4E). The distribution of the NX-02 strain was relatively dispersed in the adhesion stage (Figure 4B), and its cell gathered in some areas forming tiny colonies and organized mature biofilms. The results showed that both NX-01 and NX-02 strains could form biofilms. The biofilm formed by NX-01 strain was multilayer and denser than that of NX-02 strain, which was consistent with the results of the biofilm formation ability test (Figures 4C,D).

### 3.2. Different drug sensitivity between NX-01 and NX-02

The sensitivity results of two MG stains isolated from Ningxia to 17 kinds of antibiotics showed that the order of sensitivity of NX-01 and NX-02 to six antibiotics was similar, which from strong to weak is tetracyclines, quinolones, macrolides, pleulopleuloides, aminoglycosides, and lincomycin (Table 4). The minimum inhibitory concentration of the NX-01 strain was 2 μg/mL, and that of the NX-02 strain was 0.25 μg/mL. The NX-02 strain was more sensitive to antibiotics than the NX-01 strain.

**Table 4:** The drug sensitivity of various kinds of drugs on *M*.*gallisepticum*.

Antibiotic		Drug sensitivity(μg /mL)	
		NX-01	NX-02
Quinolones	Ofloxacin	32	8
	Enrofloxacin	16	4
	Norfloxacin	64	32
Pleuromulin	Tiamulin	32	16
	Valnemulin	32	8
Macrolide	Erythromycin	32	8
	Tylosin	4	0.5
	Tilmicosin	16	2
	Spiramycin	128	64
Tetracycline	Oxytetracycline	2	0.5
	Tetracycline hydrochloride	2	0.25
	Doxycycline	16	2
Lincomycin	Lincomycin	128	128
Aminoglycosides	Gentamicin	4	4
	Spectinomycin	64	32
	Kanamycin	64	64
	Streptomycin	64	64

### 3.3. NX-01 and NX-02 harbors MG genomic charcteristics

DNA of NX-01 and NX-2 stains were, respectively, extracted and tested the integrity. All DNA sample for sequencing were not degrade in 0.7% nucleic acid gel electrophoresis.

The genome-wide of two MG strains was sequenced using the Illumina HiSeq platform, and the total bases count of NX-01 and NX-02 strains were 1,563,147,314 and 1,184,091,000 bp, and the genome coverage was above 1,000× (Table 5). Compare and evaluate the assembly results after the initial assembly using software SPAdes, NX-01, and NX-02 strains were assembled to obtain 40 and 33 nodes, respectively. The average length of assembled sequences of them is 24608.33 and 28679.33 bp. The maximum length of a single sequences of MG strains NX-01 and NX-02 strains is 147,070 and 17,76,641 bp, respectively. The N50 lengths of them are 67,869 and 101,937 bp. NX-01 and NX-02 strains has been deposited in NCBI (Bio project PRJNA972534 and PRJNA972540).

**Table 5:** Results of 2 MG genome assembly statistics.

Genome assembly statistics	NX-01	NX-02
Total bases count (bp)	1,563,147,314	1,184,091,000
Genome coverage	1,696	1,251
Node	40	33
Average length of assembled sequence (bp)	24,608.33	28,679.33
Maximum length of a single sequence (bp)	147,070	177,641
N50 (bp)	67,869	101,937

The genome-wide of the two MG strains showed that the genome size of NX-01 and NX-02 strains were 0.98 and 0.94 Mb, respectively (Table 6), and the GC content of their genome were 31.43 and 31.46%. The predicted CDS numbers in the genomes of NX-01 and NX-02 strains were 814 and 1,548 respectively, and predicted rRNAs were 2 and 3, respectively, and both had the tRNAs of 3.

**Table 6:** Basic information of the genome-wide of 2 MG isolates.

Characteristic	NX-01	NX-02
Bp total length	984, 333	946, 418
G + Content(%)	31.43	31.46
Gene number	814	1,548
CD number	870, 411	677, 124
rRNAs number	2	3
tRNAs number	33	33
Repeat region count	0	222
CRISPR	59	31

Annotation results of NR database showed that more than 90% of the sequenced genes of the two isolates strains were derived from genome of reported MG, which proved that both of them were MG.

### 3.4. Functional genes differ between NX-01 and NX-02 genomes

**Figure 5:**
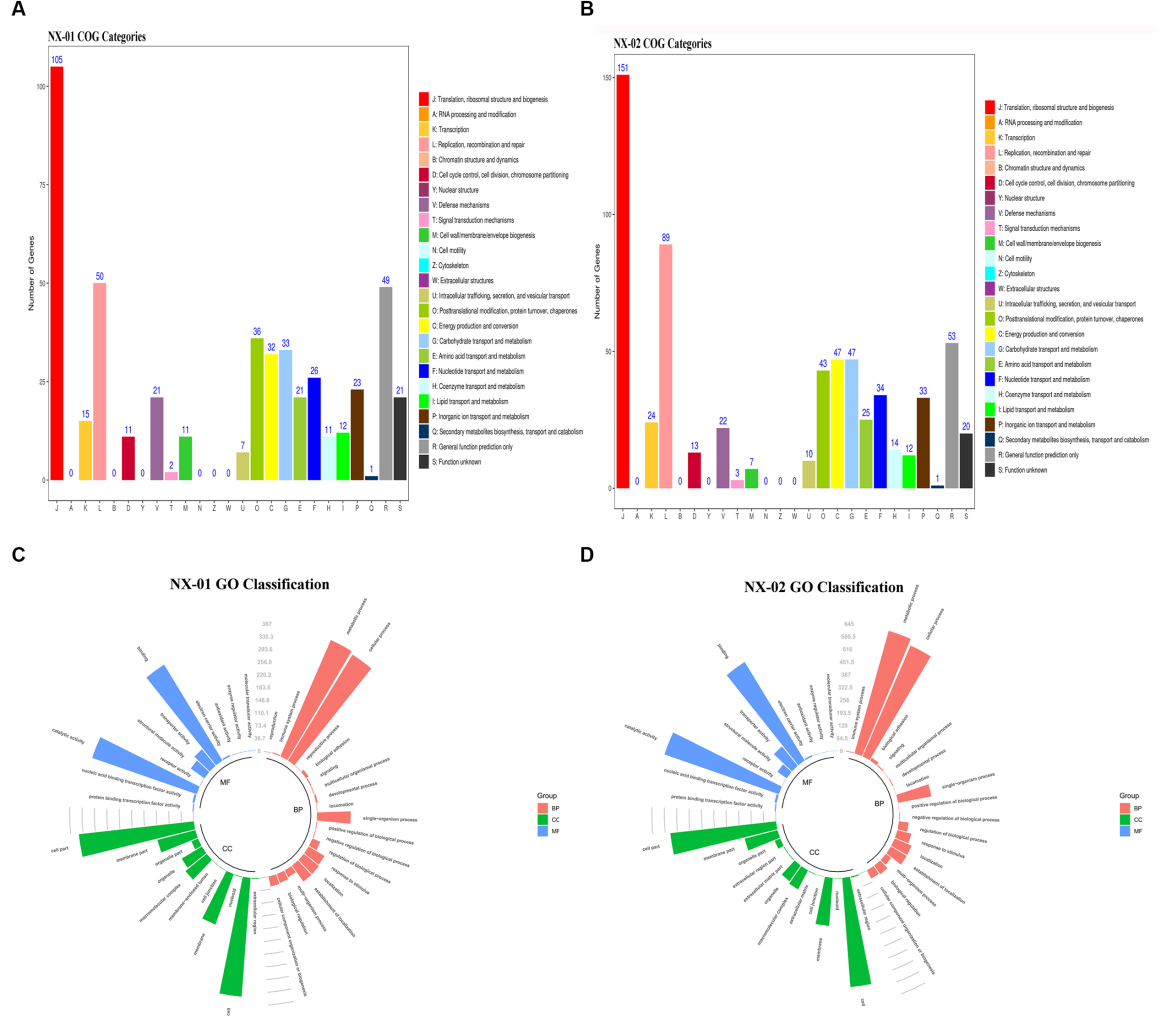
Annotation statistics of the genomes of *Mycoplasma gallisepticum* strains NX-01 and NX-02 in databases. **(A)** Functional annotation results of COG database gene of *M*. *gallisepticum* strains NX-01. **(B)** Functional annotation results of COG database gene of *M*. *gallisepticum* strain NX-02. **(C)** GO database gene classification annotation results of *M*. *gallisepticum* strain NX-01. **(D)** GO database gene classification annotation results of *M*. *gallisepticum* strain NX-02.

The functional annotation of COG genes showed (Figures 5A,B) that the number of functional genomes of NX-01 and NX-02 strains were 487 and 648, respectively. The predicted types of functional genes of these two MG strains were identical, whereas the number of certain functional genes differed significantly. The number of the two functional genes, including ribosome structure and biogenesis, and DNA replication and repair, was 40 less in the NX-01 strain than in the NX-02 strain. The number of cell wall or membrane or envelope-related genes of NX-01 strain was higher than that of NX-02 strain. The number of other functional genes of NX-01 was less or equal to that of NX-02. S-adenosylmethionine synthetase and methionyl tRNA synthetase were annotated in the genome-wide of NX-01 and NX-02 strains, both of which are biofilm-related enzymes. A biofilm-related protein, adhesin P1 precursor, was only noted in the genome-wide of the NX-01 strain.

GO database annotation showed (Figures 5C,D) that the functional genes of the two MG strains could be divided into biological process, molecular function, and cell component. NX-01 and NX-02 strains annotated 537 and 947 functional genes, respectively. The same gene may have multiple functions, the total number of genes of NX-01 and NX-02 strains involved in biological processes were 781 and 1,920, involved in molecular function were 1,127 and 1,431, involved in cell components were 1,133 and 1,785, respectively. The most annotated genes of two MG strains,in biological processes category belong to metabolic processes, including 367 genes in NX-01strain and 645 genes in NX-02 strain. The most annotated genes of two MG strains in the category of molecular functions belong to catalytic activity, including 327 genes in NX-01strain and 629 genes in NX-02 strain. The most annotated genes of two MG strains in the category of cell components belong to cells and cell parts, including 343 genes in NX-01 strain and 554 in NX-02 strain. These results indicated that the number of functional genes of NX-01 was significantly different than that of NX-02.

**Figure 6:**
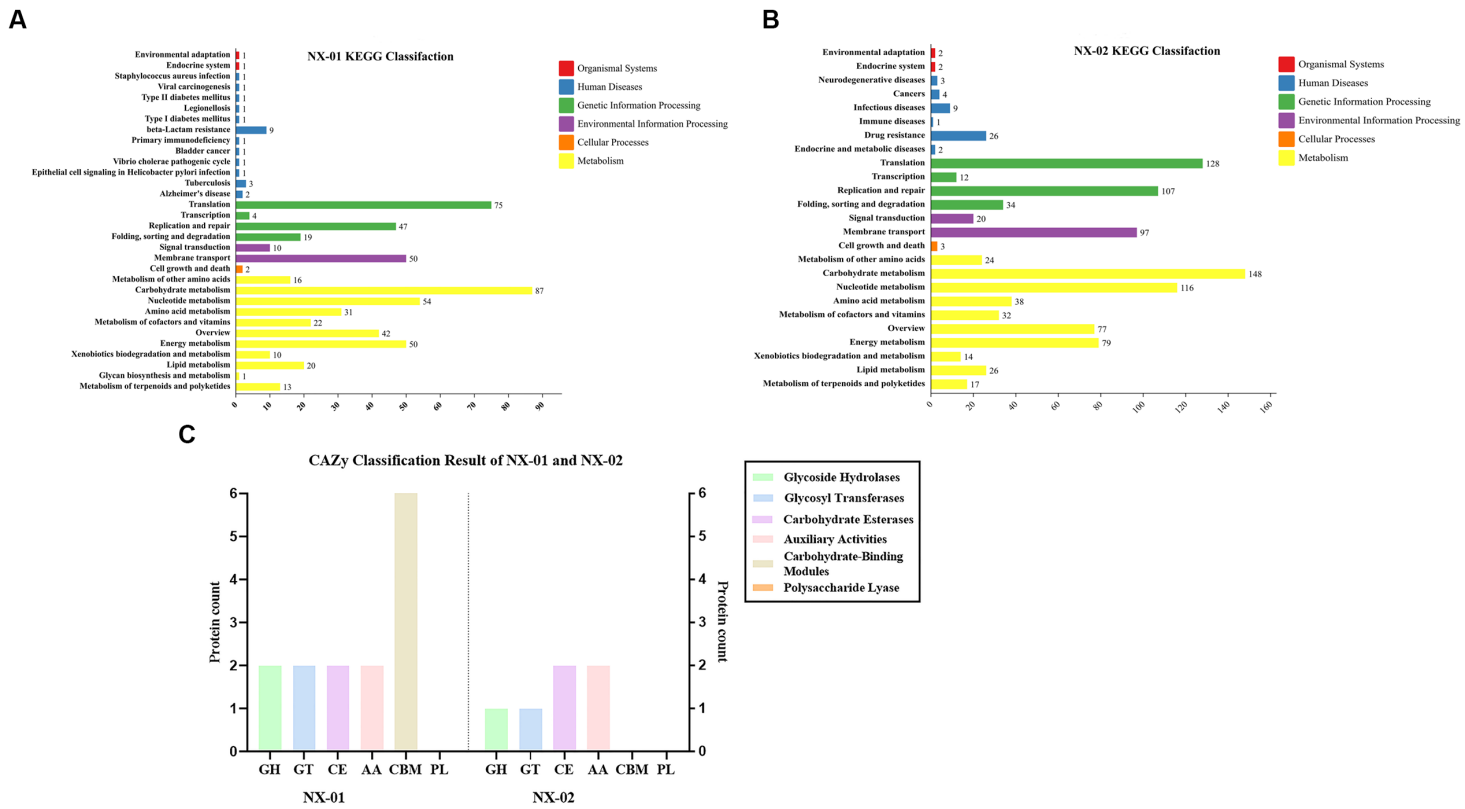
**(A)** Annotation results of KEGG database gene function classification of *Mycoplasma gallisepticum* strain NX-01. **(B)** Annotation results of KEGG database gene function classification of *M*. *gallisepticum* strain NX-02. **(C)** The CAZy database gene annotation results of *M*. *gallisepticum* strains NX-01 and NX-02.

The results of KEGG database annotation results showed that the genes of two MG strains could be divided into five branches, including cell process, environmental information processing, genetic information processing, metabolism, and organism system (Figures 6A,B). The gene number of the NX-01 strain was 302, and that of the NX-02 strain was 502. Metabolism accounted for the highest proportion of genes in the two strains. Metabolism-related genes of NX-01 strain account for 62.3% of the total genes, including energy metabolism, cofactor, and vitamin metabolism, while which of NX-02 strain lack glycan biosynthesis and metabolism genes. The number of metabolism-related genes in NX-02 strain accounted for 55.9% of the total genes. The genes related to genetic information processing accounted for 26.1% in NX-01 strain compared 27.5% in NX-02 strain. Cell process genes account for the least proportion of total genes, with only 0.3% of genes in NX-01 and 0.1% in NX-02. Five genes, *oppA*, *oppD*, *pdhA*, *eno*, and *msbA* were annotated in the environmental information processing pathway of two MG strains. Seven genes, *manB*, *pdhB*, *pdhC*, *pdhD*, *RelA*, *deoA*, and *gapA*, were annotated in the metabolic pathway. One genes, *ropS*, was annotated in the basic human pathway. All of these genes are biofilm-related gene, and among them, *PDH*, *eno*, and *gapA* participate in multiple pathways at the same time.

The annotation of the VFDB database showed that NX-01 strain has 18 species virulence factors while NX-02 has 22 species (Table 7). NX-01 strain is characterized by Cytadherence organelle, MntA transporter, and RelA virulence factor. Seven toxic factors, including Clp-type ATPase chaperone protein, type III secretion system ATPase VscN, ATPase EscN, and K1 capsule, were unique to the NX-02 strain. Hemolysin factor was the most annotated MG factor in these two strains, which was cytotoxic to various cell types. From the number of noted virulence factors, it was found that the MG strain NX-02 had more virulence factors than the NX-01 strain, which may lead to stronger virulence of the MG strain NX-02 than NX-01.

**Table 7:** Notes on the VFDB database of MG NX-01 and NX-02.

Virulence factors	Genes number	
	NX-01	NX-02
Capsule	2	2
Chu	0	2
ClpC	1	2
ClpE	1	2
Cya	2	4
Cytadherence organelle	1	0
Cytolysin	2	3
FbpABC	2	3
Hemolysin	10	8
HitABC	1	5
HSI-I	2	3
Hsp60	1	1
K1 capsule	0	1
LplA1	1	2
MgtBC	0	1
MntABC	1	0
MsrAB	1	2
Pyochelin	4	7
RelA	1	0
Shu	0	2
T3SS1	0	1
T6SS	1	2
T6SS-1	0	2
TTSS	0	3
Yersiniabactin	6	8

The annotation of the CARD database showed seven kinds of drug-resistant genes in the *M*. *gallisepticum* strain NX-01 and 14 kinds in the MG strain NX-02 (Table 8). Quinolone resistance genes is the most frequent occurring drug resistance gene in two MG strains, which NX-01 strain had 20 quinolone resistance genes, and NX-02 strain had 28. Additional resistance genes were also detected, among which the immunogenic protein *EF-Tu* accounted for the largest proportion which range from 77.7% to 87.5%. Others genes including specific lincomycin resistance factor *ImrD*, macrolide resistance factor *MacB,* aminocoumarin resistance factor *alaS*, coumarin resistance factor *novA*, rifamycin resistance factor *rpoB*, and streptomycin resistance factor *rpsL,* occurs in the NX-02 strain. The results showed that NX-02 strain annotated more drug resistance factors than NX-01 strain, and they may have different degrees of resistance to antibiotics.

**Table 8:** Notes on the CARD database of MG NX-01 and NX-02.

Antibiotic type	Gene name	Number of genes	
		NX-01	NX-02
Quinolones	ParC	2	2
	parE	7	10
	gyrA	5	5
	gyrB	6	11
Lincomycin	lmrD	0	1
Macrolides	MacB	0	1
Aminocoumarin	alaS	0	1
Coumarins	novA	0	1
Rifamycins	rpoB	0	2
	rpoC	1	1
Streptomycins	rpsL	0	1
Others	EF-Tu	7	7
	MsbA	1	1
	dfrE	0	1

The annotation of the CAZy database showed (Figure 6C) that *M*. *gallisepticum* strain NX-01 was annotated to five carbohydrate enzymes (gene number 14), including glycoside hydrolase (GH), glycosyltransferase (GT), carbohydrate esterase (CE), helper activity enzyme (AA), and carbohydrate-binding module (CBM). NX-02 strain had one less carbohydrate-binding module (CBM) and six genes. The number of related enzymes annotated by NX-01 strain was higher than that of the NX-02 strain, especially the carbohydrate-binding module, which may indicate different metabolic capacities.

### 3.5. NX-01 and NX-02 belongs to a divergent clade

**Figure 7:**
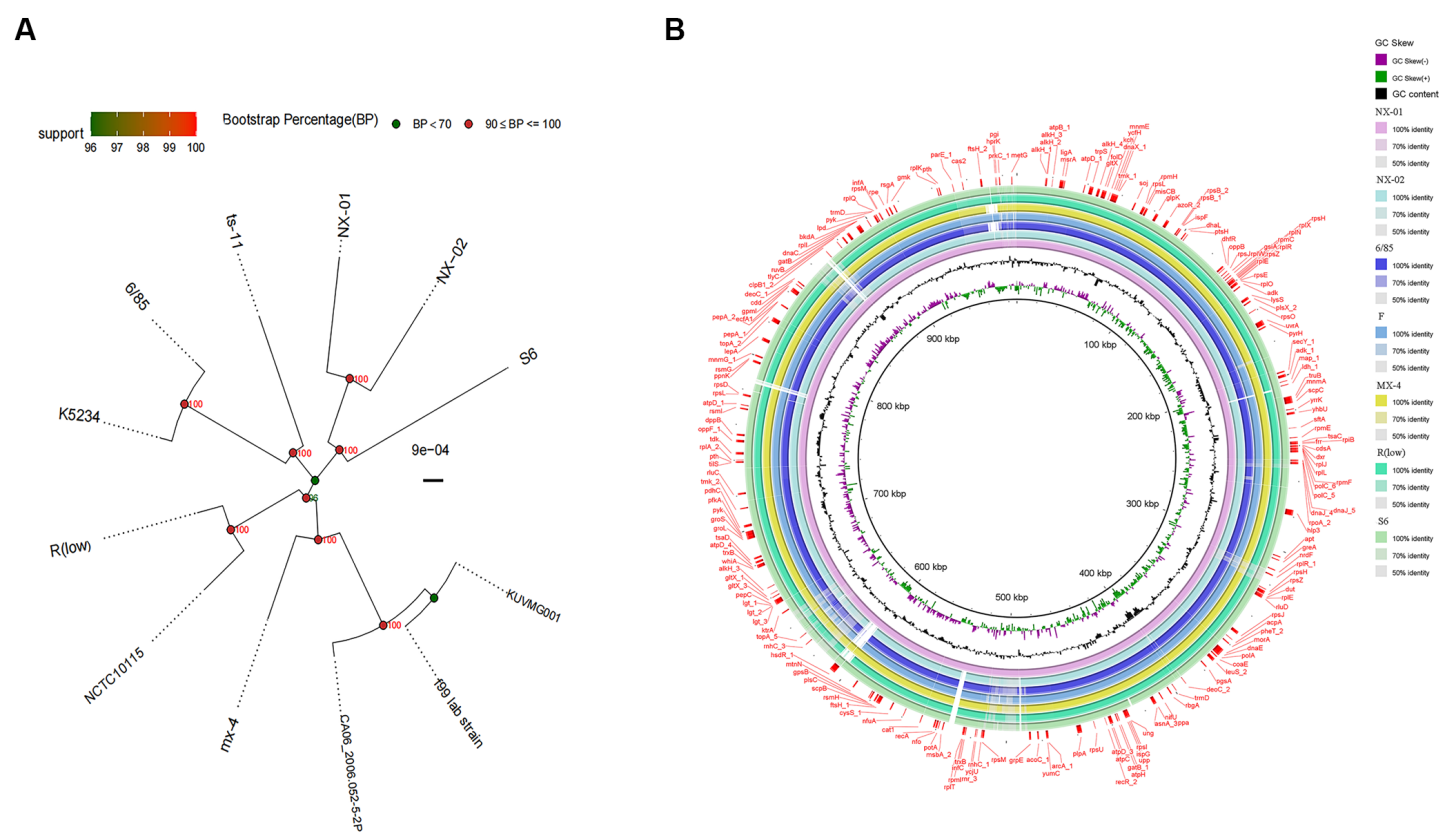
**(A)** Phylogenetic tree based on different MG genome-wide sequences. NX-01 and NX-02 strains had the closest homology and were in the same group. It was furthest related to *Mycoplasma gallisepticum* strains KUVMG01 and faa lab. **(B)** Genome circle of seven MG strains. On laps 1, 2, and 3 (from inside out), the GC skew is black. Green is skew+ and purple is skew−. In circles 4–10 are the full genomes of seven MG strains, namely *M*. *gallisepticum* strains NX-01, NX-02, 6/85, F, MX-4, R(LOW), and S6.

A phylogenetic tree based on genome-wide construction is shown in Figure 7A. NX-01 and NX-02 strains had the closest homology and were in the same group. They were slightly distant from other MG strains, such as S6, ts-11, 6/85, R(low), NCTC10115, and so on, and were on different evolutionary branches. They were related to *M*. *gallisepticum* strains mx-4 and CA06_2006-052-5-2p, and furthest related to *M*. *gallisepticum* strains KUVMG01 and faa lab. The results showed that the 12 MG strains formed three large branches without clear distinctions based on geographic location. The two Ningxia MG isolates were closely related to *M*. *gallisepticum* Russian strain S6, suggesting that the two Ningxia MG isolates and MG strain S6 may derive from a common ancestor.

The genome-wide circle map of the seven strains of MG (Figure 7B) showed that the genome-wide of NX-01 strain was significantly different from that of the other six strains. *Mycoplasma gallisepticum* strain NX-01 had fewer gene deletions and had the least difference from *M*. *gallisepticum* Russian strain S6. The MG gene differed less between NX-02 and the other five strains were less than NX-01. Compared with NX-01 strain, NX-02 strain had little genetic difference in MG with five strains from other regions. In addition, NX-02 strain has less genome-wide differences from F and R (low) strains in the United States. Five major differences were found in the comparative genome of seven MG strains, which were 490–500, 525–540, 600–615, 782–792, and 882–892 kb fragment regions. The results indicated that the genetic information of MG isolates from Ningxia had rich regional specificity.

### 3.6. Evidence of differences of NX-01 and NX-02 with other MG epidemic strains

The two MG were from Ningxia were different from the five endemic *M*. *gallisepticum* strains [6/85, F, MX-4, R(low), and S6] in five regions of the world, and the two MG isolates were different from *M*. *gallisepticum* strain F, but little from *M*. *gallisepticum* strains R(low) and S6.

**Figure 8:**
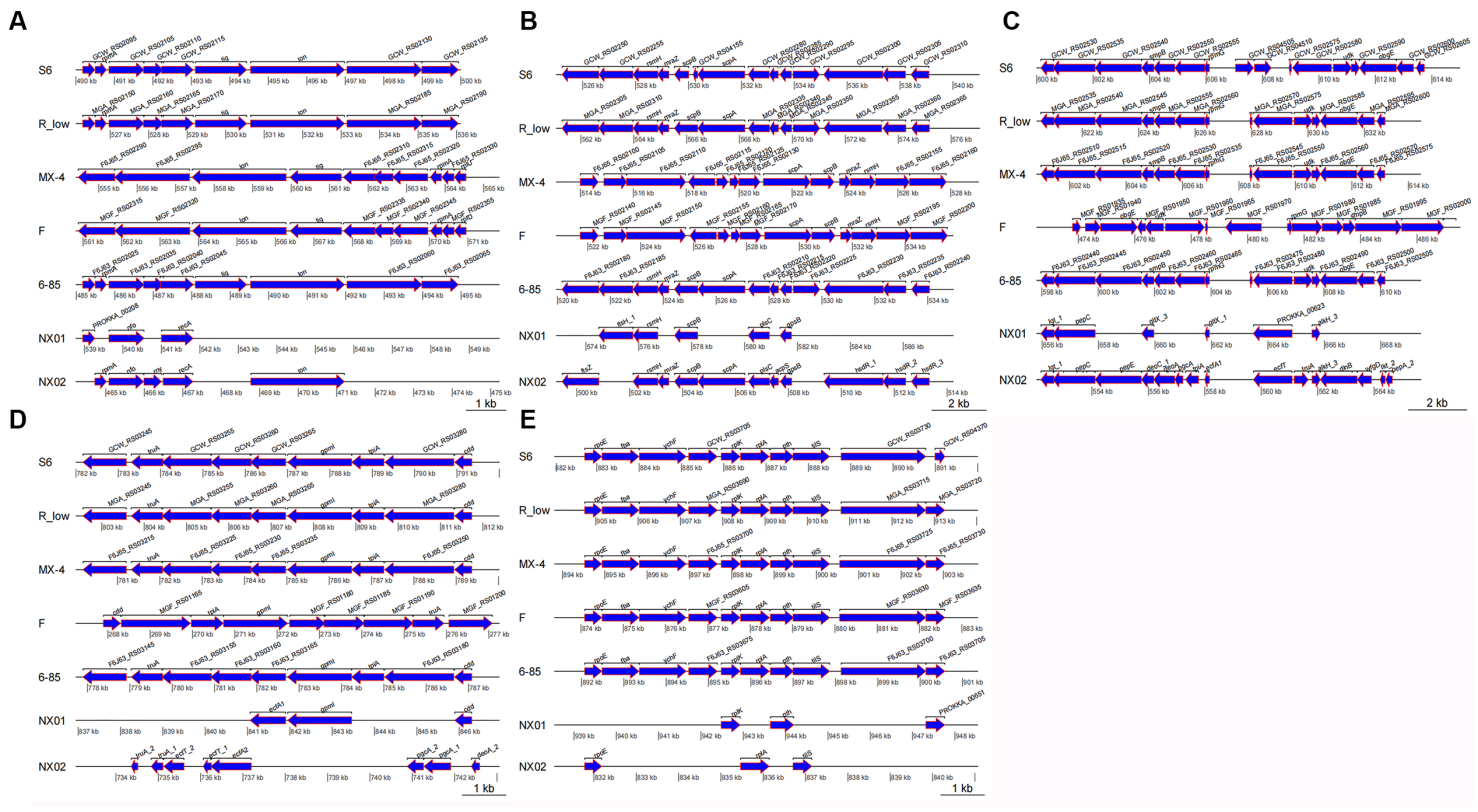
**(A)** Differences of 490–500  kb in seven MG genomes. **(B)** Differences of 520–540  kb in seven MG genomes. **(C)** Differences of 600–615 kb in seven MG genomes. **(D)** Differences of 782–792 kb in seven MG genomes. **(E)** Differences of 882–892 kb in seven MG genomes.

In the fragment region of 490–500 kb (Figure 8A), the NX-01 and NX-02 strains showed more long fragment deletion than the other five strains. In addition, NX-01 and NX-02 strains showed translocation of MG recombinase Polymerase *recA*, *rpmA*, and *nfo* compared with other five MG strains. The direction of NX-01 and NX-02 strains were consistent with S6, R(low), and 6/85 gene fragments. Compared with MX-4 and F strains, NX-01 and NX-02 strains had similar inversions of larger gene segments.

In the 520–540 kb fragment (Figure 8B), the two MG strains still had longer fragment deletion than the other five MG strains, and the same genes, such as *rsmH*, *scpB*, and *plsC*, were displaced. *Mycoplasma gallisepticum* NX-02 strain-specific *fssZ* can cause host immunogenicity. Two MG strains were in the same direction as *M*. *gallisepticum* strains S6, R(low), and 6/85, and the whole segment was inverted with *M*. *gallisepticum* strain MX-4 and F.

The 600–615 kb sequences were compared (Figure 8C). Besides removing longer fragments in two of the MG strains compared to the other five, the NX-01 strain is distinguished by the absence of *pepE*, *geoA*, and *pgcA* genes as opposed to the *M*. *gallisepticum* strain NX-02. Notably, the *pepE* was associated with the adhesion process of mycoplasma. *Mycoplasma gallisepticum* strain NX-02 had more *deoA*, *pgcA*, and *tpiA* genes than the other six strains. The two MG and *M*. *gallisepticum* strain F showed an inversion of the entire segment, and the direction of the gene segments of the other four MG strains was the same.

The 782–792 kb sequences were compared (Figure 8D), which revealed found that NX-01 strain had more deletion of MG fragments than the other six strains. NX-01 strain showed more ecfA1 gene than the other five MG isolates from different regions, and the similar genes *cdd* and *gpml* were displaced. The *ecfT*, *pgcA,* and *deoA* genes were unique to the NX-02 strain, and the similar gene *truA* was translocated. The direction of the segments of two MG strains was consistent with that of 6/85, R(low), and S6 strains, and the whole segment was inverted with F strain.

In the 882–892 kb difference region (Figure 8E), the direction of MG gene fragments in two strains was consistent with that in the other five strains. However, fragment deletion was the largest among the five different regions. The same genes *rplK*, *pth,* and *rpoE* of the two MG strains and the other five MG strains were shifted to different degrees. The length of the *tilS* in *M*. *gallisepticum* strain NX-02 was shorter and more translocated than in the other five MG strains.

## 4. Discussion

Due to the absence of cell walls and defense mechanisms, mycoplasma typically has a limited lifespan in the external environment (Justice-Allen et al., 2010). The formation of biofilms helps mycoplasma survive in the environment outside of its host (Beko et al., 2022). Thus far, only a limited number of studies have demonstrated the ability of certain *Mycoplasma gallisepticum* strains to develop biofilms, and subsequent investigations in this area are needed (Perez et al., 2020). The present study measured the biofilm-forming ability of five strains of MG isolated from chicken farms in Ningxia, China. Our findings revealed that the MG strains could create biofilms. Yet, proficiency in doing so exhibited notable variations. NX-01 strain was found to have the strongest biofilm formation capacity, while NX-02 strain had the weakest biofilm formation ability.

At the same time, scanning electron microscopy also confirmed that *M*. *gallisepticum* strain NX-01 could form a more mature and dense multilayer biofilm. On the other hand, the MG strain NX-02 could only form flaky biofilm clusters in some areas.

Biofilms provide a “safe haven” for persistent bacteria to escape antibiotics, dramatically increasing antibiotic resistance (Cepas et al., 2018; Yan and Bassler, 2019). In the drug sensitivity test of two MG strains, the MG strain NX-01, which exhibited robust biofilm formation, displayed greater antibiotic resistance than the NX-02 strain, thus demonstrating weaker biofilm formation. In general, both strains exhibited poor drug sensitivity. Currently, there is no globally accepted criterion for antimicrobial resistance in MG. Nonetheless, our results showed that the two Ningxia MG isolates had a certain degree of insensitivity to antibiotics commonly used to treat mycoplasma, which was especially evident for the *M*. *gallisepticum* strain NX-01, probably due to its strong biofilm formation capacity.

Two MG strains were predicted to contain multiple quinolone-resistant genes, such as *Parc*, *ParE*, *gyrA*, and *gyrB*. The drug sensitivity test also verified that the two MG strains were not highly sensitive to norfloxacin, which may be related to the frequent use of quinolone antibiotics in treating MG infection. Two MG-specific *RecA* genes were found in the comparative genome, and they mainly mediated SOS response. The SOS response induces resistance to antibiotic agents by repairing DNA damage caused by antibiotic agents. Quinolones are effective inducers of SOS expression(Pribis et al., 2019). The drug sensitivity test also verified the insensitivity of the two MG strains to quinolones. The NX-02 strain exhibited more specific resistance genes, such as *lmrD*, *MacB*, and *alaS,* among others, than the NX-01 strain. Despite this, the NX-02 strain demonstrated greater overall sensitivity in the drug sensitivity test than the NX-01 strain. It was observed that both biofilm and drug-resistance genes impacted the drug resistance of the bacteria. However, the mechanism underlying this phenomenon should be further explored.

By genome-wide analysis, 10 genes related to biofilm formation were predicted in both MG strains: *ManB*, *oppA*, *oppD*, *PDH*, *eno*, *RelA*, *msbA*, *deoA*, *gapA,* and *rpoS*. Phosphomanose mutase (*ManB*) is major in regulating extracellular polysaccharide synthesis of *Pseudomonas aeruginosa* biofilms, designed to maintain biofilm structure and antibiotic resistance (Ghafoor et al., 2011; Rachmawati et al., 2022). *oppA* and *oppD* belong to the ATP-dependent transporter family. They exert different roles in bacterial transport and have been shown to influence biofilm formation (Lee et al., 2004; Li H. et al., 2020). *PdhA*, *pdhB*, *pdhC*, and *pdhD* were all predicted as genes encoding pyruvate dehydrogenase complex (*PDH*). *PDH* can convert pyruvate into acetyl coenzyme A, providing energy for bacterial growth and metabolism (Matic et al., 2003). Monica also found that *PDH* operons affect the formation of *Streptococcus mutans* biofilms (Busuioc et al., 2010). Vania reported that *pdhA* regulates the formation of *Staphylococcus epidermidis* biofilms (Gaio et al., 2021). Enolase encoded by eno is a key enzyme in the glycolysis pathway and a key gene in forming *Staphylococcus aureus* biofilm (Didiasova et al., 2019; Pant et al., 2022). The ribosome-related enzyme *RelA* has been shown to induce metabolic resistance in persistent biofilm cells, used to maintain bacterial biofilms and resist antibiotics (Hall et al., 2020). *MsbA* is a multidrug-resistant protein gene that can affect bacterial adhesion and biofilm formation (Brown et al., 2014; Padayatti et al., 2019). *DeoA* is one of the key enzymes used by deoxyribonucleoside. Previous studies have shown that the microcolony of Mycoplasma enters a state of energy starvation, where deoxyribonucleoside is preferred. It helps them survive under pressure (Fisunov et al., 2022). *GapA* is an essential protein for MG host cell adhesion and virulence and may be involved in biofilm formation (Goh et al., 1998; Ruger et al., 2022). Quorum-sensing phenomenon (QS) is interdependent with biofilm formation, and *rpoS* is considered the major positive modulator of QS response. Previous studies have shown that *rpoS* is closely related to the formation of multiple bacterial biofilms (Mika and Hengge, 2014; Hall et al., 2018; Zhang et al., 2021). However, the mechanism of MG biofilm reaction with QS remains unclear.

In addition, three proteins or enzymes associated with biofilm formation were predicted: Adhesin P1 precursor, S-adenosine methionine synthetase, and methionyl tRNA synthetase. In particular, the Adhesin P1 precursor was a differential protein only predicted in the genome-wide NX-01 strain. Adhesin P1 precursor is related to adhesion protein P1, the most important membrane surface adhesion protein of *Mycoplasma pneumoniae*. It was found that adhesion protein P1 was involved in the biofilm formation of *Mycoplasma pneumoniae* and *Streptococcus mutans* (Kornspan et al., 2011; Tang et al., 2016). Therefore, the Adhesin P1 precursor might be significantly related to the distinct biofilm formation capacity of the two MG strains. However, as the genome-wide study was insufficient, further verification is necessary. In both strains of MG, the genome was analyzed for the presence of S-adenosine methionine synthetase and methionyl tRNA synthetase, where the former has a crucial role in protein methylation and various metabolic pathways in bacteria, making it a significant metabolite. It is also a precursor of QS, producing signal molecules related to inducing biofilm formation(Kleiner et al., 2019; Renard et al., 2021). Methionyl tRNA synthetase is necessary for bacteria to make proteins. Ulrike’s research has revealed that this synthetase also has a crucial role in the modulation of virulence and biofilm formation in *Acinetobacter baumannii* (Blaschke et al., 2021).

The primary invasion of host cells by mycoplasma is adhesion, which also seems necessary for forming mycoplasma biofilms (Sachse et al., 1996). Genome-wide analysis also annotated two MG adhesion-related genes. For example, heat shock proteins Hsp60, *Clpc*, and *ClpE* are important virulence factors mediating cell apoptosis, adhesion, invasion, virulence, and reproduction (Garduño et al., 1998). NX-01 strain is characterized by Cytadherence organelle, which is related to Mycoplasma’s cellular adherence and invasiveness (Romero-Arroyo et al., 1999). Among the specific virulence genes of the NX-02, TTSS, T3SS, T6SS system, and K1 Capsule had a key role in host bacterial infection (Francis et al., 2001; Schwarz et al., 2010). In addition, a specific non-catalytic carbohydrate-binding module (CBM) of the NX-01 strain was discovered from the CAZy database. CBMs attach to proteins on the surface of bacterial cells and act as carbohydrate-specific adhesins (Higgins et al., 2011). The regulatory mechanisms of MG biofilms and adhesins have not yet been fully explored; however, both were found to be closely associated with severe chronic mycoplasma infection.

The comparative genome showed that NX-01 and NX-02 strains were closely related to the Russian strain S6, which is far away from MG endemic strains in other areas. The results indicated that the two MG Ningxia isolates had regional specificity. The genome-wide of the NX-01 and NX-02 strains differed from each other. It is worth noting that NX-01 and S6 strains slightly differed, just as NX-02 with F and R(low) strains. Previous research has demonstrated that S6 strain exhibits robust biofilm formation capacity, whereas F and R(low) strains exhibit relatively poor biofilm formers. In this study, the genomic association analysis of NX-01 and S6 showed that the genomes of NX-01 and S6 strains were similar in size, 984,333 and 985,433 bp, respectively, and the CG content were also similar, 31.4 and 31.5%, with both strains containing 33 tRNAs. The P1 homologous adhesion gene was discovered in S6 strain (GenBank: U44804.1). The P1 homologous adhesin gene was discovered in strain S6 (GenBank: U44804.1), and the P1 homologous adhesin gene sequence of S6 strain was compared with the P1 adhesin precursor of NX-01 strain at NCBI Blast. The results showed that the Query Cover was 85%, the E-value was 0, and the per identity was 98.28%. This indicates that the gene sequences of the two MG strains have high homology. Only the strong biofilm formation NX-01 strain was found to contain the P1 mucoadhesive precursor, and it has been shown that the P1 mucoadhesive in *Mycoplasma pneumoniae* is associated with the formation of its biofilm.

Whether these findings are related to the ability of MG biofilm formation needs to be further investigated. We found five big differences between the two MG strains and the five MG strains from other areas, which contained homologous genes and differential genes. Also, some homologous genes were inverted, which may affect the biological characteristics of MG.

## 5. Conclusion

*Mycoplasma gallisepticum* predicted key genes that have been shown to regulate biofilm formation in other strains, and these genes may also have a regulatory role in MG biofilm formation. MG biofilm formation capacity has a direct influence on antibiotic drugs. We speculated that Adhesin P1 precursor might significantly differ from MG biofilm formation ability; however, further *in vivo* verification, such as gene knockout or use of transposon-mutated mycoplasma clones, is needed. In summary, our results provide a theoretical basis for the molecular mechanism of MG biofilm formation and a potential target for inhibiting the formation of MG biofilm.

## Data availability statement

The datasets presented in this study can be found in online repositories. The names of the repository/repositories and accession number(s) can be found at: https://www.ncbi.nlm.nih.gov/bioproject/; Two genomic information involved in this paper are PRJNA972534 and PRJNA972540.

## Author contributions

SH and FY designed the research and analyzed data. XM and LW cultured *Mycoplasma gallisepticum* biofilm. LG and YG performed genome annotation. XM performed writing—original draft preparation. SH and JL wrote, reviewed, and edited the manuscript. All authors contributed to the article and approved the submitted version.

## Funding

This work is supported by the Technology Innovation Team Construction Project of Ningxia Hui Autonomous Region (Grant No. 2022BSB03107), the School-Enterprise Joint Innovation Project of Yinchuan, Ningxia (Grant No. 2022XQ009), and the Demonstration Base Construction Project of Production-Education Integration Graduate Student Joint Training of Ningxia University.

## Conflict of interest

LG was employed by Ningxia Xiaoming Agriculture and Animal Husbandry Co., Ltd.

The remaining authors declare that the research was conducted in the absence of any commercial or financial relationships that could be construed as a potential conflict of interest.

## Publisher’s note

All claims expressed in this article are solely those of the authors and do not necessarily represent those of their affiliated organizations, or those of the publisher, the editors and the reviewers. Any product that may be evaluated in this article, or claim that may be made by its manufacturer, is not guaranteed or endorsed by the publisher.
